# Food Preferences of Winter Bird Communities in Different Forest Types

**DOI:** 10.1371/journal.pone.0053121

**Published:** 2012-12-31

**Authors:** Swen C. Renner, Sofia Baur, Astrid Possler, Julia Winkler, Elisabeth K. V. Kalko, Paul J. J. Bates, Marco A. R. Mello

**Affiliations:** 1 Institute for Experimental Ecology, University of Ulm, Ulm, Germany; 2 Harrison Institute, Sevenoaks, Kent, United Kingdom; 3 Departamento de Biologia Geral, Instituto de Ciências Biológicas, Universidade Federal de Minas Gerais, Belo Horizonte, Minas Gerais, Brazil; California State University Fullerton, United States of America

## Abstract

Food availability for forest birds is a function of habitat type, forest management regime, and season. In winter, it is also impacted by variations in the weather. In the current study we assessed the food preferences of wild bird populations in two types of forest (spruce and beech) during the months of November 2010 to April 2011 in the Schwäbische Alb Biodiversity Exploratory, south-western Germany. Our aim was to investigate whether local bird communities preferred fat-rich, carbohydrate-rich or wild fruits and to determine how forest structure, seasonality and local weather conditions affected food preferences. We found higher bird activity in beech forests for the eleven resident species. We observed a clear preference for fat-rich food for all birds in both forest types. Snow cover affected activity at food stations but did not affect food preferences. Periods of extreme low temperatures increased activity.

## Introduction

In winter, birds in the northern temperate zone face a reduced quantity and quality of food. Arthropod populations are greatly reduced or are inaccessible [1]. Snow cover reduces opportunities for foraging. Meanwhile, shorter days restrict the time available for accessing food sources, which are in turn patchily distributed [2]–[6]. At the same time, the birds themselves are subjected to increased energy loss owing to substantially lower temperatures [7]–[11]. Wintering birds therefore need to address reduced quantity and quality of food by (1) avoiding areas of food shortage, or (2) reducing energy expenditure, or (3) optimizing foraging by specialising on high quality/energy food [8], [12], [13].

Winter food availability determines the time of egg-laying and breeding success in seasonal environments [14]. In the post-winter period, and especially during the breeding season, birds favour those habitats that offer the greatest food availability [13]–[16]. This helps them compensate for energy loss during winter. Feeding preferences change at the onset of breeding (about mid-March in our study area), because the availability of animal food, especially arthropods, increases, and energy as well as nutritional needs become higher because of breeding [3], [17]–[19].

In general, both, food availability and habitat quality are closely linked to forest structure [15], [20]. Food sources are of different quantity and quality in the beech forests as compared to those in the spruce forests [21], [22] (summarized in Table 1). During winter, the two different forest types offer different foods [6], [8], [12], [13], [21], [23]. Beech forests offer high quantities of beechnut, which are relatively fatty and moderately carbohydrate-rich [21]. During the breeding and late pre-breeding season, beech forests offer also a high diversity of arthropods. In addition, beech forests have a relatively complex structure with bushes and a diverse understory of trees [2], [24]. In contrast, spruce forests offer a high quantity of low-energy, carbohydrate-rich spruce-seeds. Spruce forests have a simpler structure with fewer plant and arthropod species in the understory. In general, beech forests offer more and higher quality food for birds making it a more optimal habitat for most species in Central Europe over all other habitats.

**Table 1:** Total mass, consistency, and energy content^a^ of food offered to the winter bird community at the Schwäbische Alb exploratory.

Food source	Sequence^b^	Type	Food mass	Lard^c^	Dish	Total mass	Carbohydrates	Fat	Protein	Energy [kcal]^e^
Lard^d^	1, 2	Control	0	180	23	203	0.00	100.00	0.00	902
Sun flower seeds (dried)	3	Fat-rich	400	80	23	503	20.00	51.46	20.78	584
Oat (flakes, dried)	4	Carbohydrate	400	80	23	503	69.65	8.00	13.07	399
Rowan^e^	5	Fruit	200	50	23	273	20.30	2.00	1.50	99
Peanuts (crushed, raw)	6	Fat-rich	400	80	23	503	16.13	49.24	25.80	567
Barley (flakes, dried)	7	Carbohydrate	400	80	23	503	75.30	3.40	11.10	365
Spelt (flakes, dried)	8	Carbohydrate	400	80	23	503	70.19	2.43	14.57	338
Spruce seeds	n/a	n/a	n/a	n/a	n/a	n/a	n/a	35.1^f^	n/a	650^g^
Beechnut (dried)	n/a	n/a	n/a	n/a	n/a	n/a	33.50	50.00	6.20	576

All units in grams [g], otherwise stated.

a Adopted from [21] for 100 g [02 May 2012].

b We assigned sequence numbers for each food type on each feeding station (dish placeholder) to facilitate observations in the field.

c Added as binding agent to prevent food disappearing from dish through wind or other incidents.

d Lard made of pork (*Sus domesticus*).

e Reduced mass to fit volume (not mass) of rowan into the food dishes; values adopted from [52] for 100 g.

f Percentage of lipids [22].

g Adopted from [22].

The current study is important because although there has been considerable previous research on similar issues, most has focused only on urban areas or open landscapes or have concentrated on particular seasons. Relatively few studies have specifically addressed the effects of forest type and structure on bird feeding preferences during the pre-breeding season, despite the biological importance of this period in the annual cycle of birds [14]. Most cafeteria trials in forests have been carried out during the breeding season, aiming at testing the influence of habitat quality on breeding success and nestling growth [25], [26] or on time of breeding [27]. The current study is likely the first that integrated the two factors of weather and forest management and has extended from the winter season to the onset of the breeding season on feeding preferences of birds.

The aim of the current study was to investigate the food choice of birds in two different types of highly managed Central European forest stands during winter. These were beech and spruce forests respectively. We determine which food types are reduced under natural conditions during winter and whether forest stand type (as approximation for forest management), temperature or snow cover is the most important factor for influencing the food choice of birds.

It was assumed that birds would choose –if unlimited access to artificial food supplies in cafeteria trials is given– especially those food types which are reduced under natural conditions during winter. Therefore, if high energy food is missing in nature at that time, birds will particularly focus on this food supplement in cafeteria feeding trials.

We predicted that birds that stay in the breeding area during winter should prefer food types that are optimal for increasing the energy intake, especially fat-rich, high-energy food. We did not predict changes in food preferences (food preference = activity at a specific food source) from November to February, as fat-rich animal-based and/or plant-based food should always be the preferred food. We predicted a decrease in activity at food stations in spring (around mid March), when natural food resources become more abundant again [6], [8]. Temperature should affect food preferences; during extreme low temperatures birds should prefer fat-rich over carbohydrate-rich food and fruits, in order to level out energy loss due to lower temperature. In addition, snow cover should increase activity at food stations, because birds find less food on the snow covered ground and branches. Finally, we expected specialization in feeding preferences at the community level (i.e., different species should prefer different food types) to be higher in beech forests than in spruce forests.

## Results

**Figure 1:**
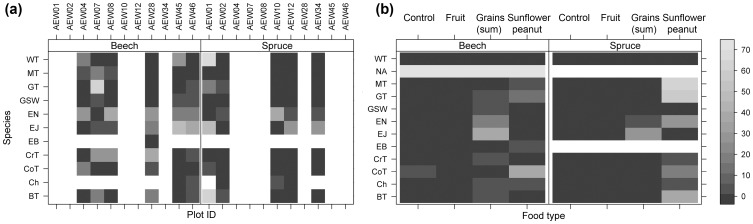
Activity of birds in forest types per species (a) for plots and (b) for food types. Any gray shaded area (scale on right hand side) indicates activity ln(*A*); white indicates zero counts for plot. Species codes: BT–Blue Tit; Ch–Chaffinch; CoT–Coal Tit; CrT–Crested Tit; EB–Eurasian Bullfinch; EJ–Eurasian Jay; EN–Eurasian Nuthatch; GSW–Great Spotted Woodpecker; GT–Great Tit; MT–Marsh Tit; WT–Willow Tit.

We observed 11 bird species (*S*; Table S1) with a total of 8,507 counts (*N*) in 280 h of observation (sum of observation hours, number of observers and number of camera traps) over 56 days of observation. Great Tits (*Parus major*) were the most frequently observed species accounting for almost half of all counts, followed by Willow Tit (*Poecile montanus*), Eurasian Nuthatch (*Sitta europea*), Coal Tit (*Periparus ater*), and Blue Tit (*Cyanistes caeruleus*) each with ≥827 *N*/*S*. Eurasian Jay (*Garrulus glandarius*), Crested Tit (*Lophophanes cristatus*), Marsh Tit (*Poecile palustris*), Great-spotted Woodpecker (*Dendrocopos major*), Chaffinch (*Fringilla coelebs*), and Eurasian Bullfinch (*Pyrrhula pyrrhula*) were observed less frequently at the feeding stations with ≤413 *N*/*S* (Figure 1a).

### Habitat and Food Preferences

We observed birds for 146 h in beech and 134 h in spruce forest. Activity depended strongly on species in each of the two habitat types: Each species and the whole community (in terms of species numbers) had twice-higher activity in beech forest as compared to spruce forest (ANOVA: activity ∼ forest type, df: 1/5026, F = 15.1, p<0.001; Figure 1a). Only Crested Tits (42.4%) had higher activity in spruce forests.

We found an effect of habitat type on food preferences. Overall activity showed a significant bias towards fat-rich food (Figure 1b). However, relative food preference did not differ significantly in both forest types. Forest type was the most important explanatory variable for activity (ANOVA: activity ∼ forest type * food type; forest type: df: 1/5020, F = 17.6, p<0.001; food type: df: 3/5020, F = 269.1, p<0.001).

### Effects of Temperature and Snow Cover on Food Preference

**Figure 2:**
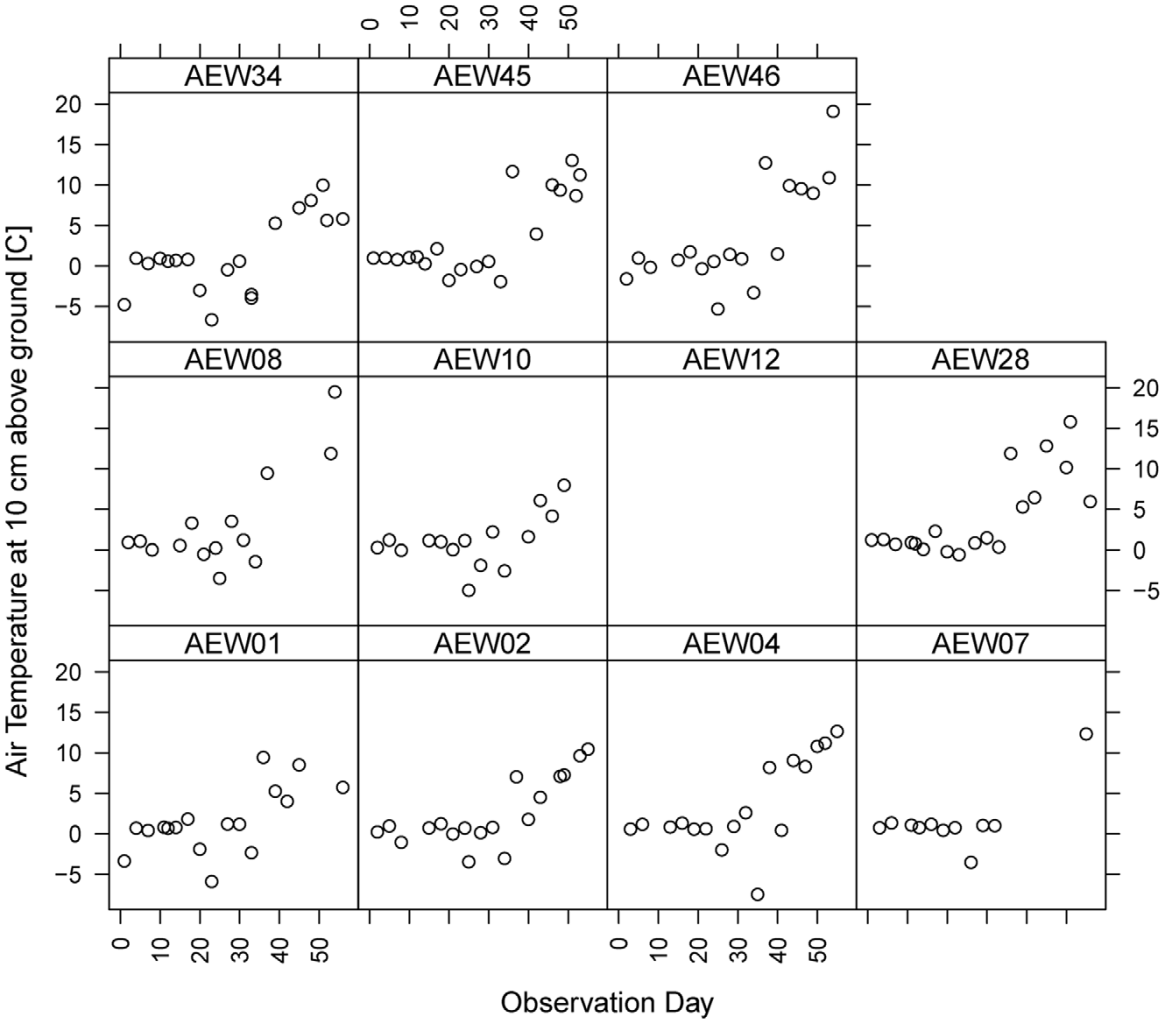
Temperature as measured at 10 cm above ground at each plot (within 50 m of feeding stations) during the observational hour per observation day (given in sequence from 1^st^ to 56^th^ day on *x*-axis. No data available for AEW12 for the observational period.

Temperature and snow cover affected activity. During the 56 days of study, the recorded air temperature at 10 cm above ground varied from −8.16 C to +19.12 C (Figure 2). In addition, 31 out of 56 days of observation had a complete snow cover in the study plots. Activity of the bird community was the same in beech and spruce forests in terms of snow cover (activity for days without closed snow cover: 333 *vs.* 257, and closed snow converge: 380 *vs.* 287). However, snow cover in general had an effect on the bird community's activity, as days with partial snow cover had significantly less activity than days with complete or no snow cover (Table 2). Activity was positively associated with snow cover at the feeding stations (ANOVA: activity ∼ snow, df: 2/1243, F = 2.54, p = 0.079; posthoc test: complete snow cover: T = 1.137, p = 0.26; partial snow cover: T = 2.068, p = 0.039).

**Table 2:** Full generalized linear mixed effects models (Poisson) fitting activity of birds at feeding stations as response to temperature (Air temperature (min), Air temperature (max), Air temperature at 10 cm), snow cover (snow), and forest structure (Roughness, Understory, Islands, Stem Zone, Euphotic Zone, and Forest Height).

			Weather				Forest structure							Model averaging			
	R code reference	Model	Air temperature (min)	Air temperature (max)	Airtemperature at 10 cm	Snow	Roughness	Understory	Island	Stem Zone	Euphotic Zone	Forest Height	Food type	K	AICc	Δ AICc	AICc weights
Activity	m1	Full model	*	*	*	*	*	*	*	*	*	*		18	17,107.20	n/a	0.09
	m3	Model1			*	*	*	*		*	*	*		14	17,104.78	0.00	0.30
	m2	Model2			*	*	*	*	*	*	*	*		15	17,104.97	0.19	0.28
	m1	Model3	*	*	*	*	*	*	*	*	*	*		11	17,105.49	0.71	0.16
	m4	Model4	*	*	*	*								9	17,105.65	0.87	0.14
	m5	Model5			*	*								9	17,105.65	0.87	0.20
		Food type^a^			*	*	*	*		*	*	*	*				

Shown are the full model and all models based on AIC-scores and 95% confidence interval; model probabilities sum to 0.9.

a as Model1 but including Food Type as fixed factor in further candidate models.

Species level response to snow cover diverged between species. European Jays (73.4% of counts with snow, 25.9% without snow cover, remainder with partial snow cover) and Willow Tits (88.5% and 11.5% respectively) had higher activity with snow cover, while Coal Tits (4.2% and 89.5% respectively) and Marsh Tits (0.0% and 72.8% respectively) had higher activity without snow or with partial snow cover only. All other species had about the same activity with or without snow cover.

Along with snow cover, we found an effect of temperature (Air temperature at 10 cm; Figure 2) on activity. While the temperature increased with the onset of March in the study area (observation day 36 onwards in Figure 2), activity remained about the same level during the entire study period in relation to temperature (compare Figure 1a).

Joining all analyses in a unified model to test which variable affected activity the most, we found that snow cover, temperature (Air temperature at 10 cm) and habitat type were always important determinants of bird activity, but food type not (GLMM models listed in Table 2, parameters of best supported model listed in Table 3).

**Table 3:** Summary of generalized linear mixed model fit by the Laplace approximation for full model (m1/Full Model in Table 2) with food type^a^, Plot ID, Observation Hour, Species Codes, and Forest Type as random factors.

Fixed effects	Estimate	Std. Error	z-value	p	Significant code
(Intercept)	1.8348	0.3183	5.76	<0.001	***
Air temperature (min)	−0.7170	1.1356	−0.63	0.528	
Air temperature (max)	0.4362	1.2626	0.35	0.730	
Air temperature at 10 cm	0.4708	2.3117	0.20	0.839	
Partial snow cover	0.9815	0.3862	2.54	0.011	*
Complete snow cover	0.2955	0.1067	2.77	0.006	**
Roughness	−1.2797	0.3752	−3.41	0.001	***
Understory	0.3143	0.1724	1.82	0.068	
Island	0.0984	0.0792	1.24	0.214	
Stem Zone	0.5637	0.2278	2.48	0.013	*
Euphotic Zone	1.1959	0.3921	3.05	0.002	**
Forest Height	−0.6198	0.1947	−3.18	0.001	**
**Fixed effects** a	**Estimate**	**Std. Error**	**z-value**	**P**	**Significant code**
(Intercept)	1.4692	0.2095	7.01	<0.001	***
Air temperature at 10 cm	0.0354	0.0129	2.73	0.006	**
Partial snow cover	0.9088	0.3894	2.33	0.020	*
Complete snow cover	0.2442	0.1018	2.40	0.017	*
Roughness	−1.2006	0.3761	−3.19	0.001	**
Understory	0.3095	0.1723	1.80	0.072	
Stem Zone	0.5396	0.2272	2.38	0.018	*
Euphotic Zone	1.0198	0.3812	2.67	0.007	**
Forest Height	−0.5618	0.1907	−2.95	0.003	**
Food type “fruit”	−0.2038	0.0759	−2.69	0.007	**
Food type “grains”	0.5210	0.0310	17.01	<0.001	***
Food type “sunflower and peanut”	0.9567	0.0296	32.34	<0.001	***

Significant codes: ***: p≤0.001, **: p≤0.01, *: p≤0.05.

a Food type was included initially as random factor but also as fixed factor in Model1.

### Seasonality of Food Preference

We found a clear temporal change in activity (ANOVA: activity ∼ Observation Day, df: 1/5026, F = 67.199, p<0.0001), but no change in activity in relation to forest types nor food type. Chaffinches where absent from our feeding stations prior to 26 March, 2011, and only occasionally observed thereafter.

**Figure 3:**
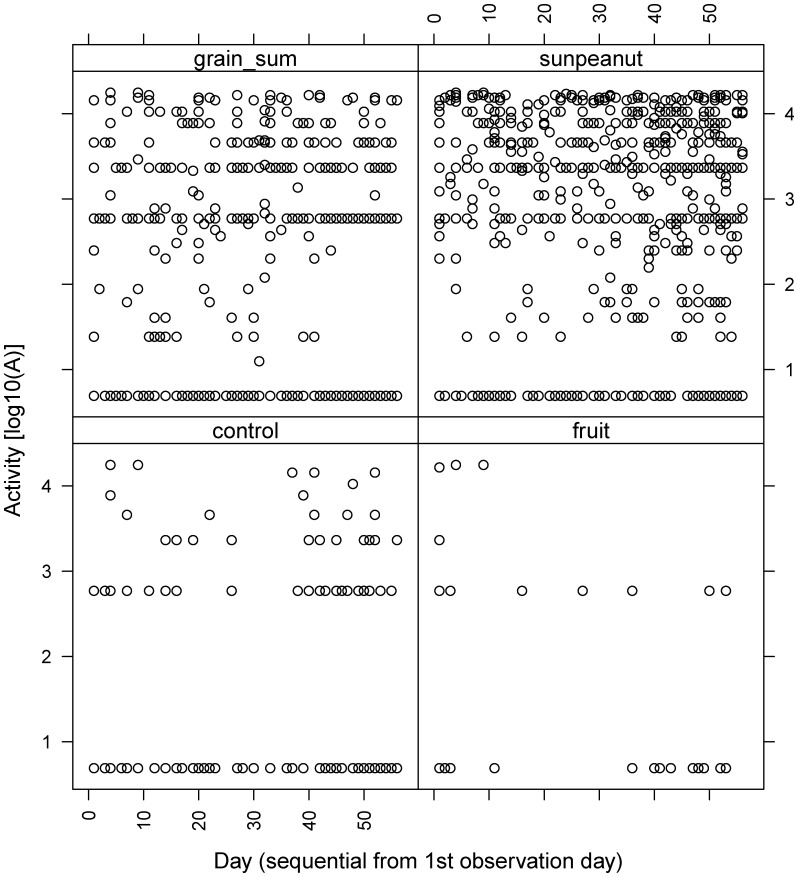
Observed activity per grouped food types during the study season from 1^st^ to 56^th^ observation day for the four grouped food types.

We did not observe any seasonal changes in food preference during our study period (Figure 3) contradicting our prediction that food sources would change at the onset of breeding in March. Activity slightly (not significantly) increases at offered grain (carbohydrate-rich), but decreases at offered sunflower and peanut grains (fat-rich) food sources towards the breeding season (mid-March to April, from observation day 36 onwards; Figure 3).

## Discussion

We found an effect of snow, temperature, and forest type on the feeding preferences of winter bird communities. In addition, birds were more active in beech forests than in spruce forests.

In general, birds respond to forest structure at different levels, from the individual to the community [1], [2], [14], [24]. If trees or parts of a forest are removed, some species may disappear locally, in order to compensate by moving elsewhere since local food availability is reduced, affecting breeding success [28]. Several bird populations decrease or increase in response to changes in forest structure and food availability [1], [24] yielding divergent responses to changes in forest structure. Under natural or natural-like conditions, bird species have species-specific preferences for particular forest structures, which are related to foraging strategies and other traits that determine bird abundance [1], [2], [22], [24], [25], [29].

We observed far more birds in beech than in spruce forests, because of differences in food availability, corroborating previous studies [1]–[3], [17], [29]. Several breeding birds that are common in the study area, such as European Blackbirds (*Turdus merula*) and European Goldfinches (*Carduelis spinus*), were absent during our study. These two species probably moved to other areas with less snow, before we began the cafeteria trials in November 2011 [25], [30]–[35], since we did not observe any individuals of European Blackbirds or European Goldfinches close to the cafeterias throughout our study.

Contrary to our predictions, we observed no difference in food preferences of birds between beech and spruce forests, since all species preferred fat-rich food in both forest types. The similarity in food preferences between beech and spruce forests is an evidence of the limited access to fat-rich food in European spruce and beech forests. In both forest types natural food had limited availability, leading to similar preference for the offered food types [34]–[36], which were mainly beechnut and spruce seeds. Deciduous forests, especially those dominated by beech trees, generally offer quantitatively and qualitatively more food for birds than spruce forests [32]. For instance, breeding success and population density of Blue Tits decrease with the percentage of conifers in their breeding habitat [37], [38]. In addition, spruce forests have almost no understory vegetation or any other plant species in higher abundance [2] and offer therefore limited food availability, represented by spruce seeds only [2]. In addition, reduced forest structure in spruce forests also decreases the availability of hideouts to escape predators.

As predicted, forest birds preferred fat-rich and to a lesser extent carbohydrate-rich food over other food types, because of their increased energy requirements in winter [39], [40]. This hypothesis is supported by the similarity between the constituents of the fat-rich food offered and the fat-rich natural food (i.e., beechnuts, spruce seeds) that are available in winter only and in limited amount [34] in forests. The preferred food of most species, regardless of forest type, was sunflower seeds and peanuts, both of which have high fat content (compare Table 1). In winter, food types with high energy content (fat-rich) are preferred by birds, because, according to the optimal foraging theory, they should maximize the difference between energy intake and expenditure. By taking fat-rich food at our feeding stations, birds profit through high energy gain with the smallest possible energy loss through food search and handling. In our experiment food search time can be disregarded, as the different food types were offered at the same time and controlled for.

The more calories a food type has, the more energy it provides for the consumer. However, it is also important to consider the interaction between carbohydrates, fat, and proteins. Carbohydrates allocate energy fast, but for a short time, whereas protein and fat offer energy for a longer time. Together, those nutrients provide the consumer with a complete supply to keep its physiological functions [41] over an extended time period and long winter nights. The preferred food, composed of sunflower seeds and peanuts, provides energy for both short and long time. In addition, fat-rich food helps build up fat reserves for the upcoming breeding season.

Oat, spelt, and barley consist mainly of carbohydrates and have just half the caloric value of peanuts or sunflower seeds. All three food types deliver short-term energy, resulting in increased need for food search. The short-term energy that oats, spelt, and barley provide indicates that birds need to invest more time in search for food, and that they need to eat more to obtain the same calories that fat-rich (animal) food would provide. Rowanberries have very little calorie content, what may explain why they were seldom consumed. The energy value of rowan is probably not high enough to transform carbohydrates into fat that could be saved for the coming breeding season. In addition, rowan might have a shape and size that are suboptimal to congest for smaller birds (as the tits of this study) making again the energy intake less favourable as with the used energy to swallow the fruit.

### Effects of Temperature and Snow Cover

Consistent with our predictions, there was an effect of snow cover on the activity of some bird species. Birds adjust individually food intake and energy saving, by using strategies such as hypothermia, hoarding and preference for specific food sources during cold and resources limited seasons [24]. Low local temperatures and snow cover change the feeding behaviour of birds [3]–[5], [11]. Small forest birds can reduced their energy loss by supplementing their energy intake when snow is covering all natural food sources, which become unobtainable and therefore unavailable even if still quite abundant below the snow [9], [39], [40]. Birds would have to change sites as an alternative (e.g., move to areas with less snow) or reduce energy loss and expenditure through other strategies to compensate for reduced availability of energy. To cope with reduced food availability, one possible strategy is to move to other areas through regular long-distance migration (at least south of the Alps) or short-distance movements (5 km), which both involve high costs for the individuals. Several bird species that breed in temperate areas move to southern Europe or Africa during winter to avoid resource scarcity (among other reasons).

Low air temperatures should increase energy requirement (e.g., found for tropical birds: [42]) to compensate for relatively higher costs of maintaining a constant body temperature. In addition, lower temperatures should trigger reduced insect activity (in March/April, at least) making further animal food resources unavailable for the birds, which in turn rely on plant-based food resources. Low temperatures are typically associated with rain or snow on the Schwäbische Alb during winter, which can be compensated through higher intake of fat-rich food.

Temperature and snow affect arthropods significantly and in Central Europe arthropods hide in safe places in winter. Animal food resources are therefore significantly reduced for birds. Animal food would easily provide fat-rich food for birds in a wintery landscape, facilitating their survival. Almost all bird species change therefore to fat-rich food. In addition, spruce forests offer more opportunities for arthropod communities to hide during winter then deciduous forests. The rough bark of spruce is a large surface with relatively safe hiding places for wintering arthropods, therefore making insects less available for birds [9]–[11]. Predation risk is probably also higher for birds at feeding stations in open beech forests then in closed spruce forests [9]–[11], [43]. Beech forests probably offer a higher quality and quantity of energy, but the energy needed for hiding from potential predators in open beech forests (compared to spruce forests) should decrease activity at feeding stations in these habitats [43]. We found the opposite: higher activity at feeding stations in beech forests, suggested that predation risk and need for high levels of energy intake are a trade off for winter birds in Central Europe.

### Conclusions

In temperate forests, bird activity is affected by snow cover and temperature. In addition, habitat type rather than food type availability affects bird activity. Our study suggests that resident winter birds are strongly affected by snow and temperature, and that food preferences change from pre-breeding to breeding. Therefore, studies on bird breeding in seasonal environments should take into account changes in food availability and food preferences.

Still missing in our framework of experiments to understand food availability are other land management strategies beyond forest management. For example, studies based on our approach could compare bird food preference (or other animals affected by seasonal environments) in grasslands or farmlands, in order to assess the relative importance of food, weather, and land management strategies for birds foraging in a constantly-changing, human-dominated world.

Most studies with feeding experiments similar to ours focused on seasonal environments; a study with a similar approach in the tropics, where seasonality is different, is currently lacking and would add valuable information on how climate zones affect food availability. Addressing whether in a less, or rather different seasonal environment (e.g., rain vs. drought rather than low temperature vs. high temperature, or natural like forest vs. oil palm plantations) birds respond to land management strategies prior to and during breeding would enhance our understanding of the importance of food availability.

## Materials and Methods

### Study Area

Our study is part of the large-scale long-term research program ‘Biodiversity Exploratories’ (details and scope are summarized in [2]), which aims at investigating the impact of forest and grassland management on various organisms and ecosystem services. Three study sites in Germany, with 50 experimental plots each, in forests of different structure and management regimes have been selected for this research program. For the present study we selected 11 experimental plots in forests of the Schwäbische Alb Exploratory in south-western Germany (approximate centre coordinates: 48.411° North and 9.497°East, 500–800 m a. s. l.).

### Forest Types and Forest Structure

Our experimental plots cover two forest management regimes, with managed forests of spruce (*Picea abies*) and beech (*Fagus sylvatica*) of different ages [2]. These 11 experimental plots have been selected based on dominant tree species (large trees with canopy cover over 70% of the experimental plot area). The experimental plots within each habitat type have supposedly similar average diameter at breast height (DBH) and age structure. While five plots have been placed in spruce forests (AEW01, AEW02, AEW10, AEW12, AEW34), six have been located in beech forests (AEW08, AEW04, AEW07, AEW28, AEW46, AEW45). The maximum distance between the plots is 20 km; plots are located in discontinuous forest patches, separated by other forest types or open landscapes.

Light Detection And Ranging (LiDAR) methods have been shown to provide continuous environmental variables that largely outcompete traditional forest type classifications which many times are also relatively coarse [36], [44]–[48]. We used LiDAR to obtain continuous environmental variables of all experimental plots during leave-on (deciduous trees) season end of July 2010. A Riegl LMS-Q 560 scanner was mounted on a helicopter flying at an altitude of 600 m. The size of the footprint diameter ranged between 20 cm and 30 cm and the sampling accuracy was 50 cm horizontally and 15 cm vertically. Based on normalized raw data and the Canopy Height model we then mathematically derived forest structure parameters, which are Roughness (vertical heterogeneity), Understory (extent of shrubs and bushes close to the forest ground), Island (canopy islands), Stem Zone (extent of the stem zone which is shows no leave, basically to open area within a forest), Euphotic Zone (leave area which resembles basically the canopy), and Forest Height (the mean height of the outer canopy height; further details of LiDAR parameters are explained in Table S1).

### Cafeteria Trials

Food types were selected to represent different amounts of energy per 100 g and had different fat to carbohydrate ratios (Table 1). Food types represented natural non-animal food of the bird species predicted for the study area and were classified as fat-rich, carbohydrate-rich, and fruit (Table 1). However, grain size of food types was similar and varied less than 1 mm in diameter (except for fruit, which were slightly larger, and control). Each food was offered in a plastic dish (hereafter ‘food dish’) in equal amounts (Table 1). Each food type was mixed with the same amount of pure lard to fix the grains to the food dish, so preventing wind or landing birds dispersing the grains. To test the effect of lard on feeding preferences, we added two control food dishes of lard to each feeding station.

At each experimental plot we placed two feeding stations (each with four food dishes), which consisted of modified, free-hanging plastic flowerpots prepared to hold the food dishes (Figures S1, S2). Each feeding station had a roof to prevent snow and rain from covering the food and also to exclude large predators. Each food dish placeholder was numbered randomly from 1 to 8, but specific numbers have been assigned to each food type (Table 1). To prevent mice and large mammals from stealing food, the feeding stations hung free (at least 1.5 m above the ground and from the next tree or branch), and the suspension ropes were blocked with free-rotating discs (Notes S1 on the procedures and methods are provided in Supporting Information). Food consumption was measured through counts of birds at feeding stations. All counts per species and time unit (60 minutes) is activity.

### Bird Observations

Observers recorded birds (counts) at feeding stations for 60 minutes in the morning (earliest start at 08: 05, latest start at 13: 35) on each observation day. The observation period started on the 30^th^ November 2010 and ended on the 8^th^ April 2011. At any given time, six camera traps (AMG Viper 3 BM Ltl-5210 were used and supported by one (until February) or two (March/April) observers. Two camera traps per plot (as each camera recorded images of four food dishes per flower pot only), recorded all birds (i.e., six camera traps at 3 plots and Possler or Winkler/Baur at other plots). Both observers and camera traps finished recording after 60 minutes simultaneously, covering four plots a day (three plots by camera traps and one by observer). We set up cameras each day to start at the same time as the observers. Each image was analyzed and birds were counted for 60 minutes. We use activity = *N* ^−h^ as our baseline unit for all comparison and analyzes, where *N* are the counted observations normalized for 60 minutes (activity), and h is the number of hours. For a single day when we observed birds for only 55 minutes, we tested the influence of the missing time and adjusted the data for 60 minutes. In case we pooled together food sources (which have been similar regarding carbohydrate or fat contents), we adjusted count values if necessary to 60 minutes counts (i.e. normalizing of data: fat-rich food was offered in two food dishes at each feeding station, grain in three, control in two, and fruit in one; Table 1).

For each day and plot we recorded start and ending time, snow cover (present/absent/partial), local temperature, and other remarks of potential interest for our study. Camera trap failures have been adjusted by later repetitions of the same plot to have always the exactly same amount of observed hours per feeding station. We obtained air temperature data for each plot as measured during the observation hour (max, min, mean of each hour when observed, measured 10 cm above ground within 50 m of the feeding stations) as available (no temperature data available for AEW12 during our study).

### Bird Activity Recorded with Cameras Traps

Each ‘count’ represented a bird observed at any feeding station. Observer counted simultaneously as the camera traps recorded birds in 5-second-intervals. Therefore we analyzed data from images and direct observations. A count was analyzed, when (1) an individual bird was detected, leaving the food station with food in the beak (we noted time/date, plot, species, food type, remarks), (2) an individual bird was observed feeding without leaving the food station (counted as 1), and (3) an individual bird was photographed at the feeding station by a camera trap (regardless of whether consuming the food or not). We did not consider counts as part of the activity, when (i) an individual bird was detected feeding several times without flying up nor without changing food dish within 5 seconds, (ii) an individual bird was detected by the camera trap in a sequence of images and it was certainly the same individual in the same food dish, (iii) an individual bird was detected at the fringe or roof of the food station, but was not feeding at any given dish (except when the bird had food in the beak), (iv) it was impossible to identify the species ('something' flying away, for instance dark image/steep shadow, only shape visible, and uncertain identification of some *Parus* species). When necessary, we corrected for different sample size (amount of plots) between habitat types in the analysis.

### Statistical Analysis

From structural and climate variables we selected models based on the information theoretic approach for activity of species at feeding stations [49] to get the minimum adequate model. We tested whether food preferences (i.e. activity at any specified food source offered) of the birds are related to forest type, temperature (Air temperature at 10 cm, minimum and maximum values), or whether snow cover affected activity of birds at the feeding stations. We used the function lm() in R for testing activity with single or combined variables. In a first step we calculated for each of our research questions an ANOVA to show response of single parameters on activity of birds at feeding stations. For seasonality, we use the Observation Day during the study as independent variable.

Finally we analyzed all variables together to establish a best fitting model explaining (with the available datasets) response of birds to temperature, snow, and forest types. Our full model included all temperature, forest types, and snow cover variables and was:

Activity ∼ Air temperature (min)+Air temperature (max)+Air temperature at 10 cm+snow+Roughness+Understory+Island+Stem Zone+Euphotic Zone+Forest Height+(1|food type)+(1|Plot ID)+(1|Observation Hour)+(1|Species Code), data = f, family = Poisson)

with activity as observed feeding event at feeding station (all variables explained in detail in Table S1). We excluded however all likely auto-correlated variables (temperature). We assumed Poisson distributions. We scaled or ln-transformed variables (however not snow, nor forest type) with the command scale() or log() in R. We used command lmer() in packages lme4() [50] on a R 2.12.0 x64 Environment [51] for all statistical analyses if not indicated differently, and lattice() [51] for plotting graphs.

## Supporting Information

Notes S1 **Methodological considerations to perform the experiments.**
(DOC)

Figure S1 **A marten, purloining sunflower seeds at plot AEW45.**
(TIF)

Figure S2 **Small mammal unsuccessfully fighting a barrier made of old Compact Discs (11 to 16 November 2010, AEW10, during nighttime).**
(TIF)

Table S1 **Metadata of the datasets used.**
(DOC)

Table S2 **Sum of all counts (N) of all observed bird species (S) in beech vs. spruce forest stands at the Schwäbische Alb exploratory from 30 November, 2010, to 8 April, 2011 (56 observational days with 202 person-camera-hours, i.e. observational hours).**
(DOC)
