# Supplementary material for: Food Preferences of Winter Bird Communities in Different Forest Types
Source: PLoS One. 2012 Dec 31;7(12):e53121. doi: 10.1371/journal.pone.0053121 (PMC3534035; doi:10.1371/journal.pone.0053121)

Notes S1: Methodological considerations

In general, we faced critical problems when establishing the feeding stations used in the cafeteria trials with the winter bird community. We addressed these problems in iterative steps during the winters 2008/2009, 2009/2010, and finally solved them and collected data in the winter 2010/2011:

(1) The largest problem were intrusive mammals (mice, squirrels, and marten), which were flexible enough to hinder our observations by removing large quantities of food. The distance between food dishes and tree branches needed to be at least 200 cm. Therefore we suspended the feeding stations with wire at least 200 cm away from any branch, blocking the access to medium-sized mammals. Unfortunately, the wires were a “stairway to heaven” for mice and small rodents (high activity of these mammals in winter was not expected, but this was proven wrong by trial, as shown in Figures A and B). To reduce predation by small mammals, we placed free circling (stopped by cork from whine bottles but still free movable) plastic discs (i.e., old CDs). Dishes were enough to stop mammals, but in some cases we had to increase disc diameter to ≥ 15 cm to fully stop robbery by small mammals. In addition, we camouflaged all discs, because the reflection in some cases affected birds negatively (own observations). Finally, we reduced predation and food purloining to minimum during our experiments (likely to zero).

(2) In the first winter, we started after a closed snow cover was established, which turned out to be a big mistake, since many birds leave the area if not sufficient food is provided. Therefore we started to feed in October each year (food mix of the food types we later provided, but not necessarily separated into the 8 different types per dish).

(3) Last but not least, many of our –for the study suitable plots– have limited access during snow cover or are restricted during winter due to timber extraction (or both) for the type of observations we intended. All plots were selected to reduce walking to less then 500 m from the next access road by snow shoes.


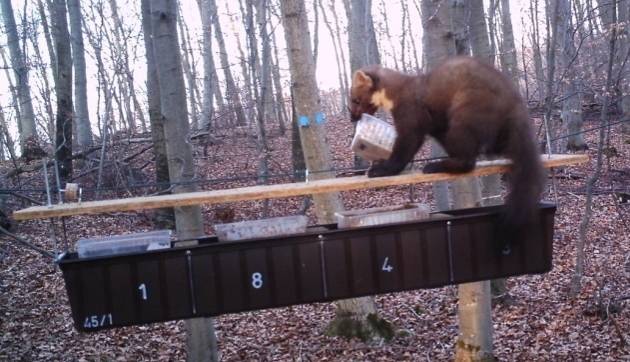
Figure S1. A marten, purloining sunflower seeds at plot AEW45

Figure S2. Small mammal unsuccessfully fighting an barrier made of old Compact Discs (11 to 16 November 2010, AEW10, during nighttime)


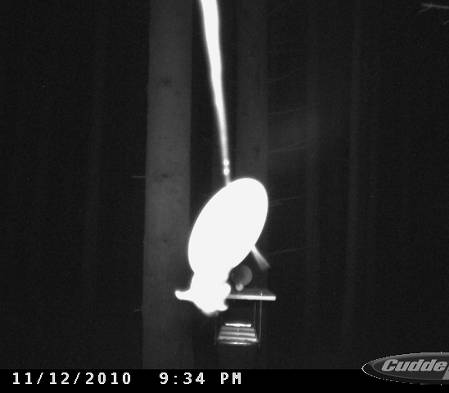

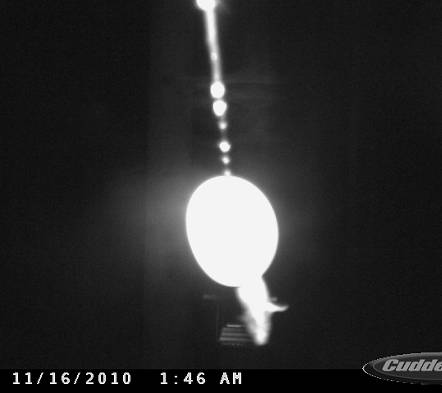

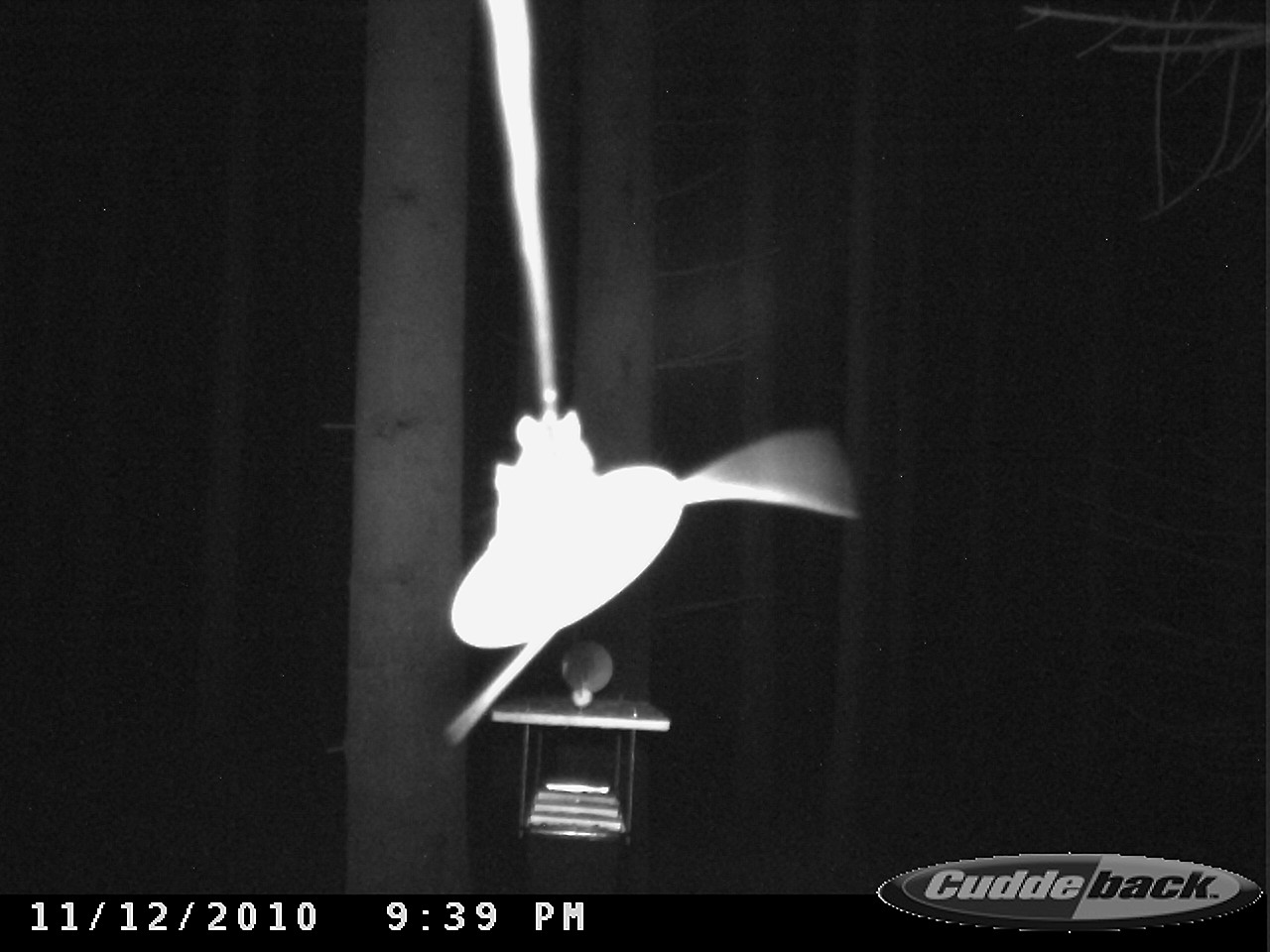

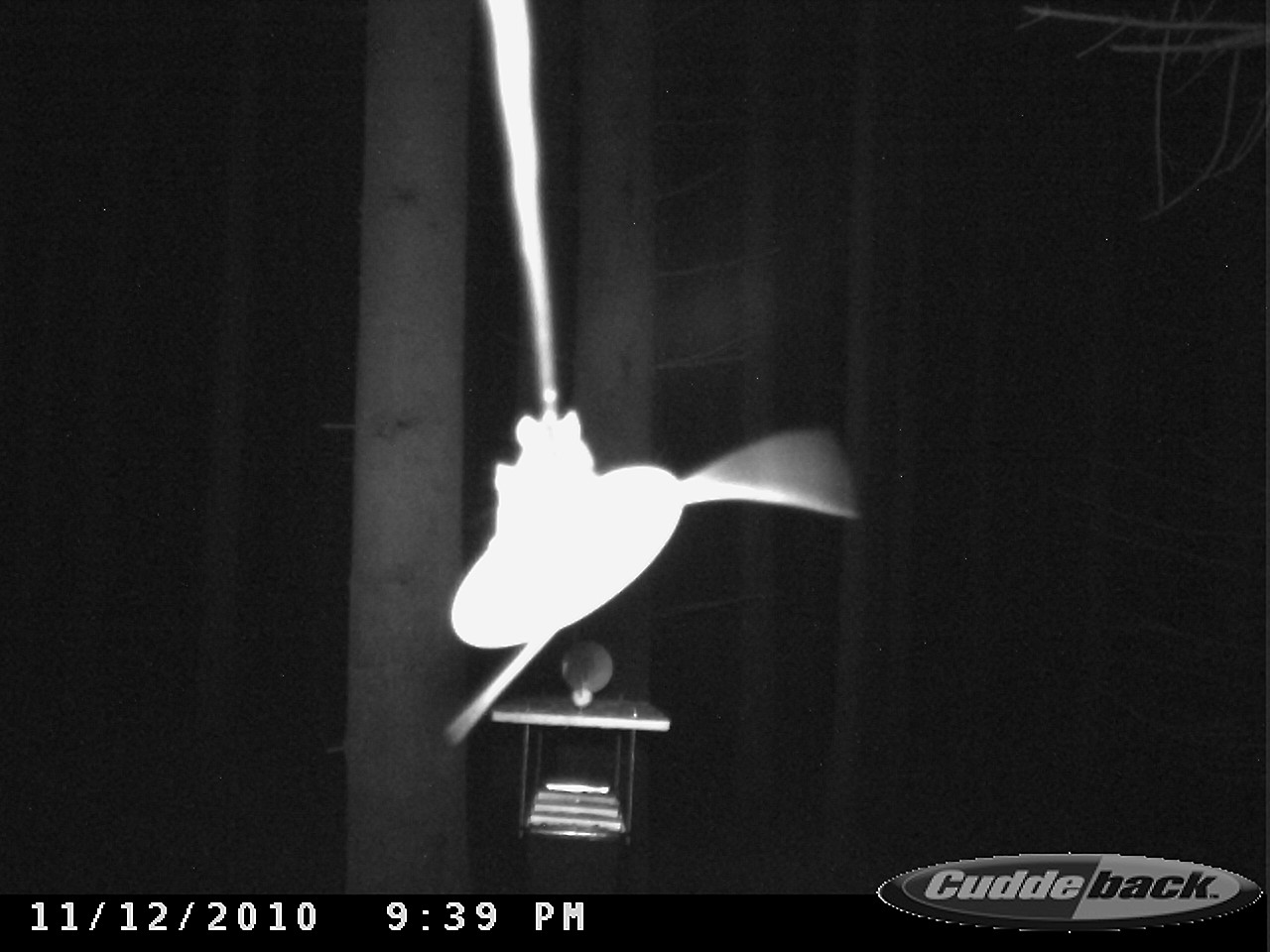

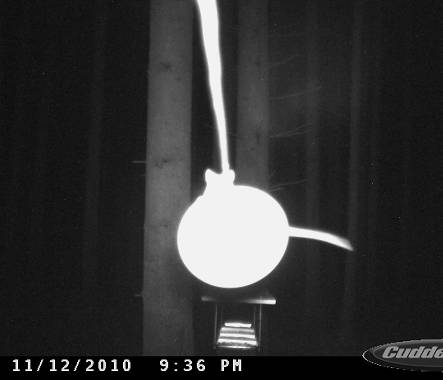

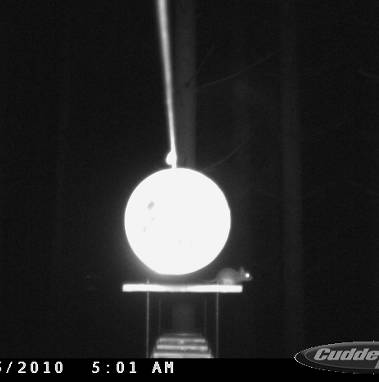

Supplement: Notes S1 — Methodological considerations to perform the experiments. (DOC) [file pone.0053121.s001.doc]
