# Supplementary figures and images for: Food Preferences of Winter Bird Communities in Different Forest Types
Source: PLoS One. 2012 Dec 31;7(12):e53121. doi: 10.1371/journal.pone.0053121 (PMC3534035; doi:10.1371/journal.pone.0053121)

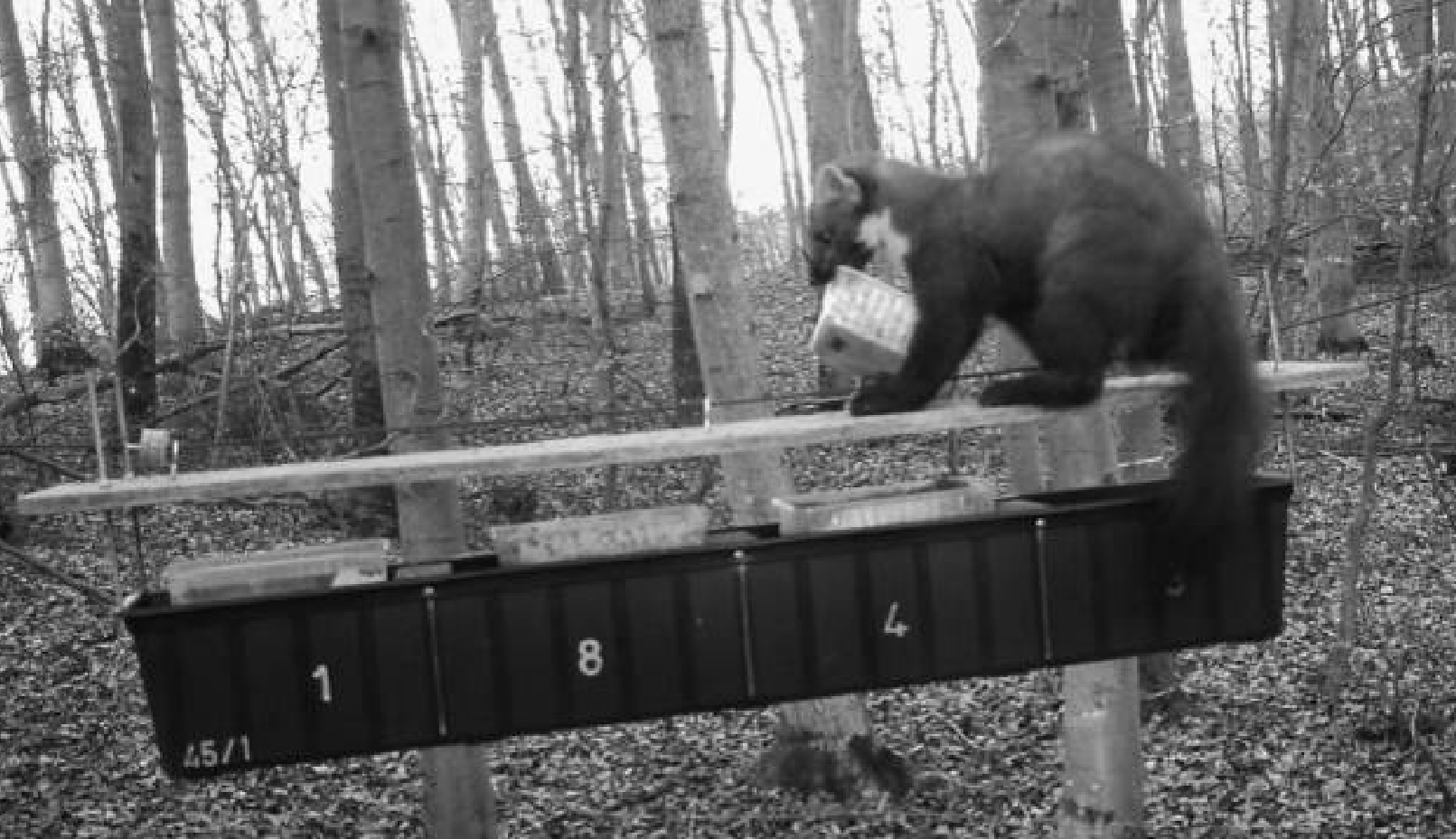

Supplement: Figure S1 — A marten, purloining sunflower seeds at plot AEW45. (TIF) [file pone.0053121.s002.tif]

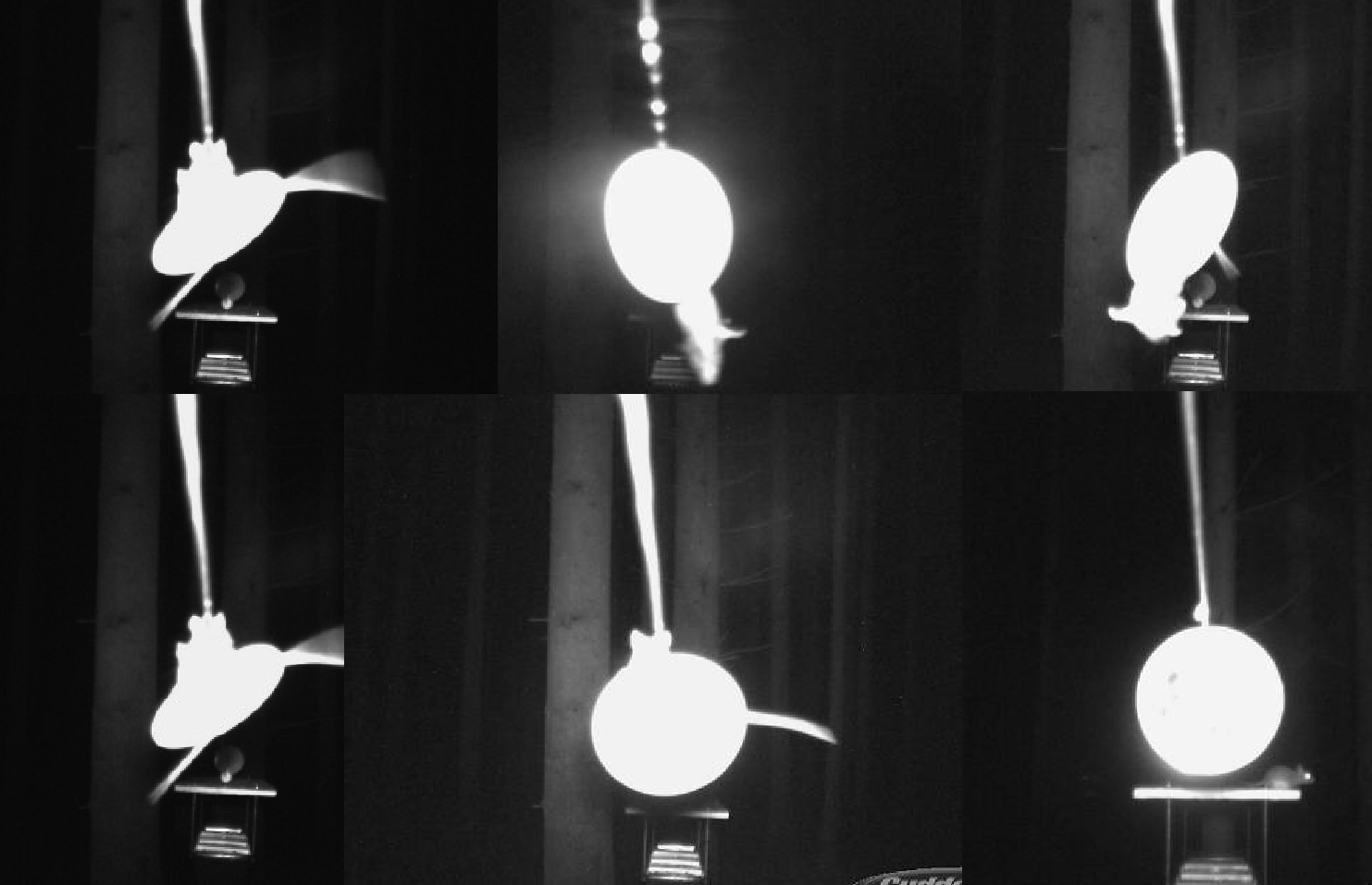

Supplement: Figure S2 — Small mammal unsuccessfully fighting a barrier made of old Compact Discs (11 to 16 November 2010, AEW10, during nighttime). (TIF) [file pone.0053121.s003.tif]
