# Supplementary material for: Food Preferences of Winter Bird Communities in Different Forest Types
Source: PLoS One. 2012 Dec 31;7(12):e53121. doi: 10.1371/journal.pone.0053121 (PMC3534035; doi:10.1371/journal.pone.0053121)
Supplement: Table S1 — Metadata of the datasets used. (DOC) [file pone.0053121.s004.doc]

Table S1. Metadata

|  | Name | Description | Unit/type |
| --- | --- | --- | --- |
| 1 | UID | Unique ID (SEQ) |  |
| 2 | Plot ID | ID of plot where observed |  |
| 3 | Observation day | Sequential day from start when observed | Integer |
| 4 | Observation hour | Sequential hour from start when observed |  |
| 5 | Forest Type | Major forest type (defined through dominant canopy tree species with  ≥ 70% cover) | class |
| 6 | Date | Date | yyyy/mm/dd |
| 7 | Time | Time when observed | hh:mm |
| 8 | Species | English species names | class |
| 9 | Species Code | Species code English names | class |
| 10 | Lard160m | Observations of species per 60 minutes at food type | Integer |
| 11 | Lard260m | Observations of species per 60 minutes at food type | Integer |
| 12 | Control60m | Observations of species per 60 minutes at food type | Integer |
| 13 | Sunflower60m | Observations of species per 60 minutes at food type | Integer |
| 14 | Peanut60m | Observations of species per 60 minutes at food type | Integer |
| 15 | Sunpeanut60m | Observations of species per 60 minutes at food type | Integer |
| 16 | Fruit60m | Observations of species per 60 minutes at food type | Integer |
| 17 | Oat60m | Observations of species per 60 minutes at food type | Integer |
| 18 | Barley60m | Observations of species per 60 minutes at food type | Integer |
| 19 | Spelt60m | Observations of species per 60 minutes at food type | Integer |
| 20 | Grain60m | Observations of species per 60 minutes at food type | Integer |
| 21 | Activity A | All observations per 60 minutes at plot | Integer |
| 22 | Air temperature (min) | Minimum Temperature measured at plot (< 50 m from cafeteria trials)  at 10 cm above ground (LT_10_MIN) | Celsius |
| 23 | Air temperature (max) | Maximum Temperature measured at plot (< 50 m from cafeteria trials)  at 10 cm above ground (LT_10_MAX) | Celsius |
| 24 | Air temperature 10 cm | Temperature measured at plot (< 50 m from cafeteria trials)  at 10 cm above ground (LT_10) | Celsius |
| 25 | Snow | Snow coverage at observation time on plot (no, partly, complete coverage) | class |
| 26 | Observer | Names of observer at plot | Integer |
| 27 | Roughness | Forest structural parameter indicating roughness | Integer |
| 28 | Understory | Forest structural parameter indicating presence of understory (shrub1) | Integer |
| 29 | Island | Forest structural parameter indicating island character of canopy (isle) | Integer |
| 30 | Stem Zone | Forest structural parameter indicating extent height) of bole zone (bolezone) | Integer |
| 31 | Euphotic Zone | Forest structural parameter indicating zone of euphotic extent (euphotic) | Integer |
| 32 | Forest Height | Forest structural parameter indicating outer canopy height (loch) | Integer |
| 33 | Food type | Food type offered (observations per 60 minutes of bird species indicated  at food type: lard1, lard2, control, sunflower, peanut, fruit, oat, barley, spelt) | class |
