# Supplementary material for: Food Preferences of Winter Bird Communities in Different Forest Types
Source: PLoS One. 2012 Dec 31;7(12):e53121. doi: 10.1371/journal.pone.0053121 (PMC3534035; doi:10.1371/journal.pone.0053121)
Supplement: Table S2 — Sum of all counts (N) of all observed bird species (S) in beech vs. spruce forest stands at the Schwäbische Alb exploratory from 30 November, 2010, to 8 April, 2011 (56 observational days with 202 person-camera-hours, i.e. observational hours). (DOC) [file pone.0053121.s005.doc]

Table S2. Sum of all counts (*N*) of all observed bird species (*S*) in beech *vs.* spruce forest stands at the Schwäbische Alb exploratory from 30 November, 2010, to 8 April, 2011 (56 observational days with 202 person-camera-hours, i.e. observational hours).

| Scientific Name | English Name | Beech (6 plots) | | Spruce (5 plots) | | Sum |
| --- | --- | --- | --- | --- | --- | --- |
|  |  | *N* | Mean *A* ± s. d. ^&^ | *N* | Mean *A* ± s. d. ^&^ | *N* |
| *Parus major* | Great Tit | 2,249 | 20.51 ± 21.44 | 1,362 | 15.32 ± 24.28 | 3,611 |
| *Parus montanus* | Willow Tit | 660 | 9.61 ± 15.44 | 362 | 6.36 ± 16.91 | 1,022 |
| *Sitta europea* | Eurasian Nuthatch | 719 | 7.43 ± 13.21 | 269 | 3.37 ± 5.4 | 988 |
| *Parus ater* | Coal Tit | 633 | 15.83 ± 14.32 | 263 | 10.96 ± 10.6 | 896 |
| *Cyanestis caeruleus* | Blue Tit | 514 | 4.77 ± 6.34 | 313 | 4.41 ± 8.51 | 827 |
| *Garrulus glandarius* | Eurasian Jay | 306 | 3.56 ± 6.87 | 107 | 1.78 ± 3.46 | 413 |
| *Parus cristatus* | Crested Tit | 143 | 1.66 ± 3.84 | 194 | 2.26 ± 3.57 | 337 |
| *Parus palustris* | Marsh Tit | 258 | 9.21 ± 15.02 | 47 | 2.94 ± 2.33 | 305 |
| *Dendroscopus major* | Great-spotted Woodpecker | 56 | 0.71 ± 1.33 | 1 | 0.02 ± 0.13 | 57 |
| *Fringilla coelebs* | Chaffinch | 30 | 4.29 ± 4.27 | 4 | 1 ± 0 | 34 |
| *Phyrrula phyrrula* | Eurasian Bullfinch | 1 | - | 0 | - | 1 |
| Total (*N*): | 11 species (S) | 5,569 | 7.85 ± 13.98 | 2,922 | 5.38 ± 13.16 | 8,491 |

^&^ Mean and s. d. take from 60-minutes time intervals (*N* adjusted for 60 minutes).
